# Opioid Peptides

**Published:** 1997

**Authors:** Janice C. Froehlich

**Affiliations:** Janice C. Froehlich, Ph.D., is an associate professor in the Departments of Medicine and Physiology/Biophysics, Indiana University School of Medicine, Indianapolis, Indiana

**Keywords:** endogenous opioids, peptides, drug interaction, neurotransmitters, opioid receptors, central nervous system, brain, neuron, biological activation, reinforcement, AOD use behavior, self administration of drugs, AOD craving, AOD sensitivity, AOD use susceptibility, euphoria, sense of pain, literature review

## Abstract

Opioid peptides produced in the body act as neuromodulators that modify the actions of other neurotransmitters in the central nervous system. By altering the electrical properties of their target neurons, thereby making these neurons more difficult to excite, opioid peptides can influence the release of various neurotransmitters. As a result of this modulation, opioid peptides can—among other functions—induce pain relief and euphoria as well as affect certain behaviors, including alcohol consumption. Alcohol can activate the opioid peptide system. This mechanism may contribute to alcohol reinforcement and excessive alcohol consumption, because agents that inhibit the opioid peptide system decrease alcohol self-administration in animals and reduce craving and alcohol consumption in human alcoholics. Moreover, a genetically determined, increased responsiveness of the opioid system to alcohol may contribute to a predisposition for alcoholism in some people.

Endogenous opioid peptides^1^ are small molecules that are naturally produced in the central nervous system (CNS) and in various glands throughout the body, such as the pituitary and adrenal glands. These peptides produce the same effects as the chemicals known as classic alkaloid opiates, which include morphine and heroin. Endogenous opioid peptides function both as hormones and as neuromodulators. Endogenous opioid peptides that serve as hormones are secreted into the circulation by the producing glands and are delivered to a variety of distant target tissues where they induce a response. Endogenous opioid peptides that serve as neuromodulators are produced and secreted by nerve cells (i.e., neurons) and act in the brain and spinal cord to modulate the actions of other neurotransmitters. (For more information on these neurotransmitters, see the related articles in this section.) Through these two mechanisms, endogenous opioid peptides produce many effects, ranging from preventing diarrhea to inducing euphoria and pain relief (i.e., analgesia).

This article reviews the physiology of endogenous opioid peptides and their interactions with other neurotransmitters. In addition, the article summarizes the interactions between alcohol and the endogenous opioid system and presents evidence that opioid peptides play a role in alcohol reinforcement. (For further information on alcohol and the opioid system, see Froehlich and Li 1993, 1994; Gianoulakis and colleagues 1996*a*; Herz 1997.)

## Physiology of Opioid Peptides

### Opioid Peptide Production

Many peptides with opioidlike effects have been found in the CNS and in peripheral tissues. Molecular biological approaches, such as recombinant DNA techniques, have demonstrated that these peptides fall into three categories—enkephalins, endorphins, and dynorphins—that are derived from three distinct precursor molecules. The active, functional opioid peptides are generated from their precursors by enzymes called peptidases, which cut these precursor molecules into smaller entities. The peptides are then modified further during posttranslational processing, which can include the addition of various chemical groups to the peptides (e.g., sugar molecules [i.e., glycosylation], acetyl groups [i.e., acetylation], phosphate groups [i.e., phosphorylation], or methyl groups [i.e., methylation]). These modifications can alter the peptides’ biological activities. Because the processing of opioid peptides from larger precursor molecules is very selective, the opioid-peptide profiles can vary among different tissue types.

So far, two of the enkephalins (i.e., leuenkephalin and met-enkephalin) and one of the endorphins (i.e., beta-endorphin) have been shown to be active in mediating alcohol’s effects.

### Opioid Receptors

To affect the functions of their target cells, opioid peptides must bind to specific molecules, or receptors, on the surfaces of these cells. The body contains several receptors that selectively recognize molecules with opioidlike structures. Three major categories of opioid receptors—mu, delta, and kappa—have been identified that differ both in their functions and in their binding characteristics. A given opioid peptide can interact with more than one type of opioid receptor. The binding of opioid peptides to these receptors initiates a series of biochemical events that culminate in various effects, including analgesia and euphoria.

### Site of Action of Opioid Peptides

#### Anatomical Distribution in the Brain

**Figure 1:**
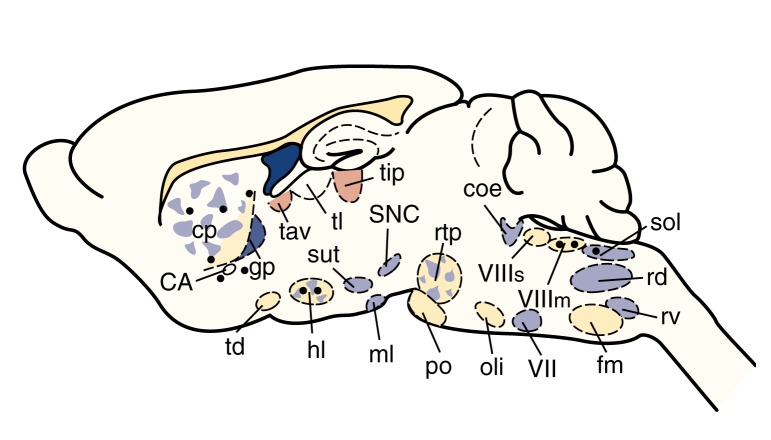
Schematic representation of a rat brain illustrating the distribution of enkephalin-containing neurons. These neurons originate in numerous brain regions (indicated by black circles) and terminate either in the same regions or in distant regions (indicated by shaded areas). NOTE: CA=commissura anterior; coe=ceruleus n.; cp=n. caudatus putamen; fm=n. paraventricularis pars magnocellularis; gp=globus pallidus; hl=n. lateralis hypothalami; ml=n. mammilaris lateralis; oli=n. olivaris; po=n. pontis; rd=n. reticularis dorsalis medullae oblongatae; rtp=n. reticularis tegmenti pontis; rv=n. reticularis ventralis medullae oblongatae; SNC=substantia nigra zona compacta; sol=n. tractus solitarii; sut=n. subthalamicus; tav=n. anterior ventralis thalami; td=n. tractus diagonalis; tl=n. lateralis thalami; tlp=n. lateralis thalami, pars posterior; VII=n. nervi facialis; VIIIm=n. vestibularis medialis; VIIIs: n. vestibularis superior. SOURCE: Adapted from Sar, M.; Stumpf, W.E.; Miller, R.J.; Chang, K.-J.; and Cuatrecasas, P. Immunohistochemical localization of enkephalin in rat brain and spinal cord. *Journal of Comparative Neurology* 182:17–38, 1978.

Not all opioid peptide-containing neurons are located in the same brain areas. For example, neurons that contain enkephalins have been found in many different brain regions, suggesting that these peptides are involved in many physiological functions (see figure 1). For example, enkephalins mediate pain perception by acting in the spinal cord and in the periaqueductal gray (PAG) region of the brain. In addition, enkephalins alter emotional responses by acting in limbic areas, such as the amygdala, and alter cardiovascular or respiratory functions by acting on autonomic nuclei in the hypothalamus and brain stem. Most enkephalin-containing neurons have short axons, indicating that enkephalins act close to their points of synthesis.

**Figure 2:**
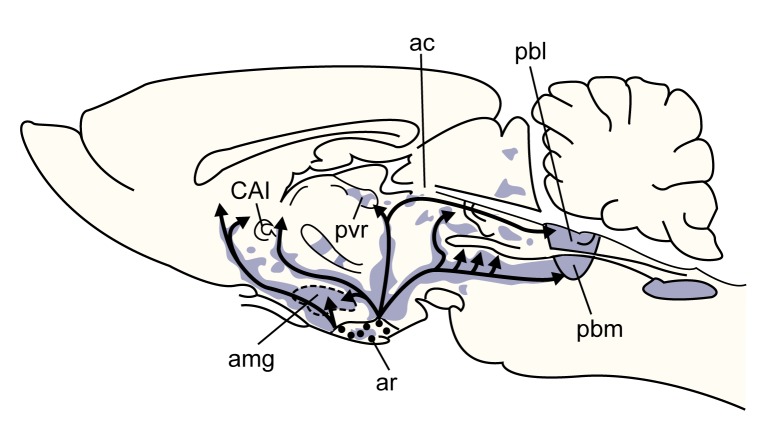
Schematic representation of a rat brain showing the distribution of β-endorphin–containing neurons. Most of these neurons originate in the arcuate nucleus in the hypothalamus. As indicated by the arrows, the neurons extend to a variety of brain regions, terminating in the shaded areas. NOTE: ac=n. amygdaloideus centralis; amg=amygdala; ar=arcuate n.; CAI=capsula interna; pbl=n. parabranchialis lateralis; pbm=n. parabranchialis medialis; pvr=n. periventricularis rotundocellularis. SOURCES: Finley, J.C.W.; Lindstrom, P.; and Petrusz, P. Immunocytochemical localization of β-endorphin-containing neurons in the rat brain. *Neuroendocrinology* 33:28–42, 1981; Konig, J.F.R., and Klippel, R.H. *The Rat Brain*. Baltimore: Williams & Wilkins, 1963; Ungerstedt, U. Stereotaxic mapping of the monoamine pathways in the rat brain. *Acta Physiologica Scandinavica* 367(Suppl.):1–48, 1971.

The distribution of neurons containing the other two types of opioid peptides—beta-endorphin and the dynorphins—is not as diffuse as that of the neurons containing enkephalins. Neurons that contain beta-endorphin are found predominately in the hypothalamus and in a region of the brain stem (i.e., the nucleus of the solitary tract) (see figure 2). The dynorphin-containing neurons are located primarily in the hypothalamus. In contrast to the enkephalin-containing neurons, those that contain beta-endorphin or dynorphins have long axons that extend to distant brain regions as well as to the pituitary gland, brain stem, and spinal cord, indicating that beta-endorphin and dynorphins act distant from their points of synthesis.

#### Anatomical Distribution in the Periphery

All three types of opioid peptides (i.e., enkephalins, endorphins, and dynorphins) are found in the pituitary and adrenal glands. Although many stimuli can induce the pituitary to release opioid peptides into the circulation, the precise function of these peptides in their peripheral target tissues is not well understood. Opioid peptides have been found in many organ tissues throughout the body, including the heart, pancreas, placenta, kidneys, and gastrointestinal organs.

### Mechanism of Action of Opioid Peptides

Once released from the neurons, opioid peptides act through opioid receptors on their target neurons to transmit messages that primarily inhibit secondary systems, such as pain perception (see below). In addition, opioid peptides can induce the following effects:

- Decrease respiration
- Stimulate or depress cardiovascular functioning, depending on the brain area where the opioid peptides act
- Decrease the movement (i.e., motility) of the muscles of the gastrointestinal (GI) tract, thereby slowing down the food’s transit through the GI tract
- Decrease susceptibility to seizures
- Induce euphoria
- Affect certain behaviors, such as the consumption of food and alcohol.

#### Pain Control Systems

Endogenous opioid peptides induce analgesia in humans and antinociception in animals.^2^ These peptides act in several regions of the CNS to mediate pain control, because antinociception is observed in animals whether endogenous opioid peptides are administered into the peripheral circulation; into spinal sites; or into various regions of the brain, such as the raphe nuclei, PAG region, or medial preoptic area.

Many events or stimuli that are experienced as painful, stressful, or traumatic can induce the release of endogenous opioid peptides. These peptides then act to make humans and animals less sensitive to noxious events by inducing euphoria and analgesia or antinociception.

Evidence suggests that acupuncture induces analgesia by stimulating the release of endogenous opioid peptides. For example, chemicals that inhibit opioid receptor function (i.e., opioid receptor antagonists) have been shown to reverse the analgesia induced by acupuncture. Similarly, the action of inactive substances, or placebos, in reducing pain may result from the placebo-induced release of endogenous opioid peptides in anticipation of pain relief. This hypothesis is supported by the fact that opioid receptor antagonists reverse the pain relief experienced with placebos.

## Interactions Between Opioid Peptides and Other Neurotransmitters

Endogenous opioid peptides are produced and often released together with other neurotransmitter molecules in the brain, pituitary gland, and adrenal gland as well as by single neurons in the central and peripheral nervous systems. For example, neurons in the carotid body simultaneously release an enkephalin and dopamine. Similarly, single neurons in the peripheral nervous system of rats simultaneously release an enkephalin and the neurotransmitter norepinephrine.

Although the function of the co-release of peptide-neurotransmitter pairs is not always clear, evidence suggests that opioid peptides can alter the release of other classic neurotransmitters. Opioid peptides generally make their target neurons more difficult to excite by increasing the voltage difference that exists between the inside and the outside of the cell (i.e., by hyperpolarizing the neurons). As a result of this hyperpolarization, the neuron’s “firing rates” and neurotransmitter release are reduced. However, if the neuron receiving the opioid peptide signal is an inhibitory neuron (i.e., a neuron that reduces the activity of the cells it affects), the opioid signal may inhibit the inhibitory neuron, thereby producing excitation (i.e., disinhibition) of a neighboring cell.

Opioid peptides have been reported to inhibit the release of acetylcholine, dopamine, and norepinephrine in both the brain and the peripheral nervous system. In addition, opioid peptides can increase as well as decrease the release of serotonin and gamma-aminobutyric acid in the brain. The integrated response to the administration of opioid peptides in a given brain region is difficult to predict, because this response depends on the excitatory versus inhibitory nature of the cells or neurons that receive the opioid-peptide stimulation. The fact that opioid peptides act as neuromodulators, altering the release of other neurotransmitters, adds another level of complexity to the neuronal networks in the brain, spinal cord, and periphery that may contribute to complex behaviors, such as alcohol consumption.

## Alcohol and the Endogenous Opioid System

Evidence suggests that alcohol-induced activation of the endogenous opioid system may be part of a neurobiological mechanism that is involved in mediating alcohol reinforcement and excessive alcohol-drinking behavior. This view is based on three lines of evidence.

The first line of evidence stems from analyses of the effects of opioid receptor antagonists on alcohol consumption. Opioid receptor antagonists compete with the endogenous opioid peptides for binding to the opioid peptide receptors, in effect displacing the endogenous opioid peptides from the receptors and thereby reversing the peptides’ effects. Thus, when opioid receptor antagonists alter a biological process, one can infer that endogenous opioid peptides are involved in that process. Both nonselective opioid receptor antagonists that affect all types of opioid receptors (e.g., naloxone and naltrexone) and antagonists that are selective for the mu and delta opioid receptor types have been shown to decrease alcohol self-administration in rodents and monkeys under a variety of experimental conditions.

Based on these findings, two double-blind, placebo-controlled clinical trials investigated the effects of naltrexone on alcohol drinking in outpatient alcoholics. These studies demonstrated that naltrexone decreased the mean number of drinking days per week, the frequency of relapse, the alcohol-induced subjective “high,” and the desire to drink. In 1994 naltrexone (Revia^TM^ or Trexan^®^) was approved by the Food and Drug Administration as a pharmacotherapeutic agent for the treatment of alcohol dependence when used as part of an appropriate plan of addiction management. Naltrexone is the first pharmacotherapeutic agent that decreases alcohol consumption by reducing the reinforcing properties of alcohol rather than by inducing illness, as does disulfiram (Antabuse^®^).

The second line of evidence suggesting that endogenous opioid peptides are involved in regulating alcohol-drinking behavior comes from neurobiological studies indicating that alcohol alters the activity within opioid peptide systems. Acute alcohol consumption or the exposure of isolated cells and tissues to alcohol increases endorphin and enkephalin gene expression in certain brain regions of rodents, increases opioid peptide release from both the brain and the pituitary gland of rodents in tissue- and organ-culture experiments, and increases opioid peptide release from the pituitary of both rodents and humans in the intact organism. Conversely, chronic alcohol administration decreases opioid gene expression in rat brain, and chronic drinking decreases beta-endorphin release in alcoholics.

The third line of evidence supporting the functional involvement of the opioid system in alcohol-drinking behavior is derived from studies indicating that both in rodents and in humans, a genetic predisposition toward alcohol consumption is accompanied by an increased responsiveness of the opioid system to alcohol. For example, in a rat strain selectively bred for high rates of alcohol consumption, acute alcohol administration produces larger increases in beta-endorphin gene expression in the pituitary and enkephalin gene expression in the brain compared with rats selectively bred for low alcohol consumption. Similarly, alcohol exposure induces a greater and more prolonged release of opioid peptides in brain tissue from alcohol-preferring mice compared with tissue from mice that do not prefer alcohol.

In humans, beta-endorphin responses to alcohol differ among subjects with and without a family history of alcoholism. Thus, alcohol consumption induces a significant increase in plasma beta-endorphin levels in subjects at high risk for the future development of alcoholism (i.e., with a family history of alcoholism), but not in those at low risk (i.e., without a family history of alcoholism). Taken together, these results from animal and human studies suggest that genetic differences in alcohol-drinking behavior may stem in part from variations in the responsiveness of the opioid system to alcohol.

## Alcohol Reinforcement and the Endogenous Opioid System

Significant experimental evidence suggests that beta-endorphin and the enkephalins function as positive reinforcers. In humans, a strong correlation exists between feelings of well-being or euphoria and plasma levels of beta-endorphin. In addition, beta-endorphin administration into the fluid-filled cavities of the brain (i.e., the ventricles) produces mood elevation. In rodents, both beta-endorphin and the enkephalins act as reinforcers, as evidenced by the fact that the animals self-administer opioid peptides and prefer locations where they have received opioid peptides in the past. Taken together, these observations suggest that greater alcohol-induced activation of the endogenous opioid system in genetically predisposed animals or humans may enhance the reinforcing properties of alcohol, thereby increasing the probability of subsequent drinking. This effect may contribute to the development of a preference for and the continued ingestion of alcohol. Enhanced responsiveness of the opioid system to alcohol may be a heritable trait associated with, and causally related to, excessive alcohol-drinking behavior.

## Summary

Research findings obtained over the past two decades have suggested that opioid peptides serve a variety of physiological functions. Moreover, alterations in endogenous opioid peptide systems may contribute to a variety of clinical conditions, including alcoholism, obesity, depression, diabetes, and epilepsy. The release of opioid peptides in response to alcohol consumption may reinforce further alcohol drinking. Moreover, enhanced sensitivity of opioid peptide systems to alcohol may contribute to a predisposition for the development of alcoholism in certain people.

## Footnotes

1 For a definition of this and other technical terms used in this article, see central glossary, pp. 177–179.

2 The term “analgesia” refers to a decrease in the subjective response to the sensation of pain in humans, whereas a reduction in the response to pain in animals is termed “antinociception.”
